# Advancements in Cardiovascular Disease Research Affected by Smoking

**DOI:** 10.31083/j.rcm2508298

**Published:** 2024-08-21

**Authors:** Miaoxin Fu, Aihua Mei, Xinwen Min, Handong Yang, Wenwen Wu, Jixin Zhong, Chunlei Li, Jun Chen

**Affiliations:** ^1^Sinopharm Dongfeng General Hospital (Hubei Clinical Research Center of Hypertension), Hubei Key Laboratory of Wudang Local Chinese Medicine Research, Hubei University of Medicine, 442000 Shiyan, Hubei, China; ^2^School of Public Health, Hubei University of Medicine, 442000 Shiyan, Hubei, China; ^3^Department of Rheumatology and Immunology, Tongji Hospital, Huazhong University of Science and Technology, 430074 Wuhan, Hubei, China

**Keywords:** tobacco, secondhand smoke (SHS), nicotine, cardiovascular diseases (CVD), atherosclerosis, coronary heart disease, hypertension

## Abstract

The harmful substances in tobacco are widely recognized to exert a significant detrimental impact on human health, constituting one of the most substantial global public health threats to date. Tobacco usage also ranks among the principal contributors to cardiovascular ailments, with tobacco being attributed to up to 30% of cardiovascular disease-related deaths in various countries. Cardiovascular disease is influenced by many kinds of pathogenic factors, among them, tobacco usage has led to an increased year by year incidence of cardiovascular disease. Exploring the influencing factors of harmful substances in tobacco and achieving early prevention are important means to reduce the incidence of cardiovascular diseases and maintain health. This article provides a comprehensive review of the effects of smoking on health and cardiovascular diseases.

## 1. Introduction

Cardiovascular diseases (CVD) stand as a primary cause of morbidity and premature mortality, resulting in 17.9 million deaths annually and accounting for 31% of the global mortality rate [1] . Smoking and various non-tobacco factors, such as metabolic and lipid-related factors, as well as hypertension, are recognized risk factors for CVD [2, 3, 4, 5]. The most prevalent and lethal form of tobacco use is smoking, with lifelong smokers losing an average of at least 10 years of life [6]. Globally, there are over one billion smokers, and tobacco-related causes account for approximately 6 million deaths annually among smokers and those exposed to secondhand smoke. If not controlled, tobacco could potentially result in one billion deaths in the 21st century [7]. Smoking can lead to a range of severe diseases, lower overall health status, impair immune function, and reduce quality of life. Research data indicate that smoking impacts not only long-term smokers but also individuals exposed to secondhand smoke in their vicinity, leading to a cascade of negative health effects [8, 9]. Smoking exerts harmful effects on numerous organ systems and is a chief culprit in diseases such as lung cancer, pneumonia, chronic obstructive pulmonary disease, head and neck cancers, urological and gastrointestinal cancers, periodontal disease, cataracts, and arthritis [10]. Furthermore, smoking is a pivotal modifiable risk factor in the occurrence of cardiovascular diseases, including coronary artery disease, stable angina, acute coronary syndrome, sudden death, stroke, peripheral vascular disease, congestive heart failure, and the initiation and progression of aortic aneurysms during atherosclerosis development.

Smoking leads to diabetes, induces insulin resistance, and concurrently increases the risk of ischemic heart disease. It also diminishes immunity and contributes to over 30 diseases, including tuberculosis [3]. Besides these, smoking also exacerbates age-related conditions such as cardiovascular diseases, atherosclerosis, and cellular senescence [11]. Both active and passive smoking confer similar harm to the cardiovascular system, particularly among individuals frequently exposed to smoke in their homes and workplaces [12, 13]. The results include decreased heart rate, systolic blood pressure, exercise tolerance, and symptomatic ischemic heart disease [14]. The morbidity and mortality resulting from secondhand smoke exposure also primarily stem from cardiovascular diseases, notably ischemic heart disease [15]. Consequently, both active and passive smoking elevate the risk of coronary artery thrombosis and myocardial infarction, with the incidence and fatality of cardiovascular diseases closely correlated to smoking history [16]. Research suggests that smoking is a primary risk factor for acute coronary thrombosis, and acute thrombosis in smokers can lead to sudden cardiac death [17]. Lv *et al*. [18] conducted a meta-analysis of 23 prospective cohort studies and 17 case-control studies, revealing that among nonsmokers, those exposed to secondhand smoke had a 1.23-fold higher risk of coronary heart disease compared to those unexposed. Smoking also influences all stages of atherosclerosis, triggering coronary artery disease, inducing ventricular repolarization anomalies, and augmenting the risk of sudden death [19, 20]. Smoking can initiate and expedite the progression of atherosclerosis by impairing vascular endothelium. Other potential mechanisms encompass endothelial injury, endothelial dysfunction, oxidative stress and damage, thrombosis, lipid abnormalities, and inflammation [21]. Hence, reducing both active and passive smoking can effectively diminish disease risk, enhance blood circulation, prolong lifespan, and alleviate stress.

## 2. Impact of Smoking on the Heart

### 2.1 Harmful Substances in Tobacco

#### 2.1.1 Role of Nicotine

Nicotine is an alkaloid extracted from the leaves of tobacco plants (Nicotiana tabacum and Nicotiana rustica) and serves as the primary addictive substance in tobacco products [22]. Nicotine also plays a major role in tobacco smoke, acting through nicotinic acetylcholine receptors (nAChRs) in neurons within the brain [23, 24]. It acutely dilates cerebral arteries and arterioles through the release of nitric oxide from nitrergic neurons, while chronically interfering with endothelial function in various vessels [25]. One gram of nicotine can be lethal to 300 rabbits or 500 mice. An injection of 50 milligrams of nicotine can be fatal in humans. Nicotine can bind to nicotinic acetylcholine receptors in various parts of the body, including the central nervous system, and exerts positive stimulating effects through neurotransmitter release, such as dopamine [8]. Nicotine addiction involves the midbrain dopamine system, contributing to reward-related sensations and associative learning in the early stages of addiction. Chronic nicotine addiction also affects homeostatic dysregulation in neurons, modulates midbrain GABAergic circuits, regulates neuronal scaffolding proteins, and alters epigenetic processes [26].

On one hand, tobacco smoke contains a high concentration of nicotine, which can activate sympathetic neurotransmission, induce endothelial oxidative stress, and lead to endothelial dysfunction and atherosclerotic lesions [27]. On the other hand, the most significant toxic compounds in tobacco-derived smoke are nitrosamines and polycyclic aromatic hydrocarbons, both known carcinogens associated with numerous complications, including various cardiovascular diseases [28]. Unique tobacco-specific nitrosamines have also been identified in the vapor of electronic cigarettes containing nicotine [29]. Nicotine nitrosation, which occurs during the use of electronic cigarettes, has been suggested in some studies to potentially contribute to the development of human lung cancer, bladder cancer, and heart disease [30]. Previous research indicates that up to 30% of global litter comprises cigarette waste. These discarded cigarette butts are non-biodegradable and harbor over 7000 toxic substances, including benzene, 1,3-butadiene, nitrosamines, N-nitrosonornicotine, nicotine, formaldehyde, acrolein, ammonia, aniline, polycyclic aromatic hydrocarbons, and various heavy metals. These toxic substances have serious detrimental effects on the ecological environment and may lead to more serious health problems such as cancer, respiratory diseases, and cardiovascular diseases [31]. Exposure to high concentrations of environmental tobacco smoke, whether through active or passive smoking, can lead to endothelial dysfunction, promote the occurrence and progression of atherosclerosis, and have a serious impact on cardiovascular health [32].

#### 2.1.2 Impact of Harmful Chemicals on the Cardiovascular System

Cigarette smoke is an aerosol containing over 4000 chemicals, including nicotine, carbon monoxide, acrolein, and various oxidative compounds. Exposure to cigarette smoke induces a range of pathological effects within the endothelium, some of which result from oxidative stress triggered by active oxygen, reactive nitrogen species, and other oxidative components present in the smoke. This exposure adversely affects control over all stages of plaque formation, progression, and pathological thrombosis. Active oxygen in cigarette smoke reduces the bioavailability of nitric oxide, leading to oxidative stress, upregulation of inflammatory cytokines, and endothelial dysfunction. The formation of plaques and the development of vulnerable plaques are also attributed to exposure to cigarette smoke, enhancing the activation of inflammatory processes and matrix metalloproteinases. Additionally, exposure to cigarette smoke leads to platelet activation, stimulation of coagulation cascades, and impaired anticoagulant and fibrinolytic activity. Many smoke-mediated prothrombotic changes are rapidly reversible upon smoking cessation [33].

The toxicological components in tobacco products, including nicotine, carbon monoxide, particulate matter, oxidants, heavy metals, phenols, and flavoring agents, can all contribute to adverse cardiovascular events [34]. Nicotine, the primary addictive substance in tobacco, causes chronic smoking to lead to desensitization and upregulation of nicotine receptors. Its sympathomimetic activity affects heart rate, myocardial contractility, vasoconstriction of skin and coronary arteries, transiently elevates blood pressure, reduces insulin sensitivity, and can potentially exacerbate or trigger diabetes, possibly leading to endothelial dysfunction [22]. Moreover, the carcinogens in tobacco can induce DNA damage, accelerate aging, and potentially increase the incidence of cardiovascular diseases in smokers [35].

Annually an estimated 6 million deaths annually worldwide are attributed to smoking-related causes, with nearly 10% of cardiovascular disease-related deaths attributable to smoking [3]. Smoking is a major contributor to cardiovascular disease and mortality. Research suggests that if trends in tobacco use, diabetes, obesity, and hypertension continue unchecked, premature deaths from cardiovascular disease will rise from 5.9 million in 2013 to 7.8 million by 2025 [36]. Long-term smoking is directly associated with cumulative cardiovascular damage, particularly among current smokers. In North Africa, the Middle East, Central Asia, sub-Saharan Africa, high-income Asia-Pacific, and Western Europe regions, smoking cessation significantly reduces premature cardiovascular mortality in male populations [36]. Reports indicate a rapid decline in cardiovascular disease incidence shortly after quitting smoking, suggesting that short-term effects of smoking may also be acutely important, possibly related to inflammation, thrombosis, endothelial dysfunction, arterial stiffness, and coronary microvascular dysfunction [37]. On the other hand, secondhand smoke contains higher concentrations of many carcinogens and toxic chemicals than the smoke inhaled by active smokers. A case-control study indicates that maternal exposure to secondhand smoke during the first three months of pregnancy may increase the risk of coronary heart disease in offspring [38]. Smoking not only contributes to chronic cardiovascular diseases but is also a leading cause of acute atherothrombotic events like stroke or myocardial infarction [33]. In summary, smoking poses significant health risks to both active and passive smokers and has severe environmental consequences. Discarded cigarette butts, secondhand and thirdhand smoke exposures have irreversible adverse effects on ecosystems and human health [39].

### 2.2 Blood Circulation and Blood Pressure

#### 2.2.1 The Impact of Smoking on Blood Circulation

The relationship between active smoking and hypertension appears to be complex and varies across studies. Some research suggests a potential link between smoking and blood pressure changes, but its long-term effects are debated and may depend on factors like smoking intensity, duration, age, and gender. For instance, studies have indicated a possible negative correlation between current smoking and certain blood pressure parameters, with smokers having similar or slightly lower blood pressure than non-smokers, possibly due to the leaner body composition of smokers [40]. Another study highlighted how smoking might induce renal damage and microvascular dysfunction, potentially exacerbating oxidative stress in kidneys and thus impacting blood pressure regulation [41, 42]. It is important to note that despite occasional reports of a negative correlation, the general medical consensus views smoking as a risk factor for hypertension and other cardiovascular diseases. These findings should be interpreted cautiously, and more research is necessary to fully understand the long-term impacts of smoking on blood pressure and overall cardiovascular health.

Whether active or passive, smoking poses significant harm to the human body. Smoking can induce changes in microcirculation, leading to severe and extensive damage to the body. Proteomic analysis of platelets has confirmed that smoking triggers various inflammatory responses, and beyond complications in large arterial atherosclerotic lesions, damage to the microvasculature can lead to organ failure [43, 44]. A study suggests that nicotine in tobacco can render rat myocardium susceptible to ischemia-reperfusion injury, increase mitochondrial reactive oxygen species generation and permeability transition, cause left ventricular dysfunction, and exacerbate the myocardial ischemia-reperfusion process [45]. Additionally, the rapid elevation of arterial nicotine concentrations stimulates autonomic nicotinic acetylcholine receptors, subsequently desensitizing them, leading to disruptions in cardiac function, systemic and uterine hemodynamics, reducing uterine-placental blood flow, and causing smoking-associated pregnancy complications and developmental impairments in pregnant women [46].

Environmental tobacco smoke is positively correlated with hypertension [46, 47, 48]. A study involving over 9000 children found that children exposed to environmental tobacco smoke in utero have a higher likelihood of developing hypertension, particularly elevated systolic blood pressure. Among them, girls whose fathers smoke are at an increased risk of hypertension when exposed to environmental tobacco smoke [49]. Individuals with significant exposure to secondhand smoke in the environment have an almost threefold increased risk of the presence of any atherosclerotic plaque, a twofold higher risk of obstructive coronary artery disease, and involvement of at least three major arteries compared to non-exposed individuals [50]. Furthermore, there is a significant relationship between the extent of exposure to a secondhand smoke environment and the number of major vessels involved, segments affected by plaques or stenosis, coronary artery calcium scores, and the percentage of segments with calcified, partially calcified, and non-calcified plaques [51, 52]. The mechanisms underlying increased cardiovascular risk due to secondhand smoke exposure are multiple and interactive, potentially leading to adverse reactions in both the cardiovascular and respiratory systems. Long-term exposure to secondhand smoke, apart from its adverse health effects, not only impacts health over the long-term and chronically but also acutely, particularly concerning ischemic heart disease [53, 54]. Therefore, both active smoking and exposure to the secondhand smoke environment not only affect normal blood circulation but can also significantly increase the incidence and severity of coronary artery calcification, leading to more severe cardiovascular diseases.

#### 2.2.2 Interaction Between Smoking and Lipid Abnormalities

Recent studies have emphasized that the complex interaction between smoking and lipid abnormalities is a key risk factor for coronary artery disease (CAD). Research focusing on this interaction indicates that smoking significantly influences lipid levels. Smokers exhibit elevated levels of triglycerides (TG) and reduced levels of high-density lipoprotein cholesterol (HDL-C), both crucial factors in the development of coronary heart disease. This study also highlights that these lipid changes partially mediate the relationship between smoking and CAD risk, with the TG/HDL-C ratio serving as a significant mediator. This finding underscores the importance of monitoring lipid profiles in smokers for assessing CAD risk [55].

**Fig. 1.**
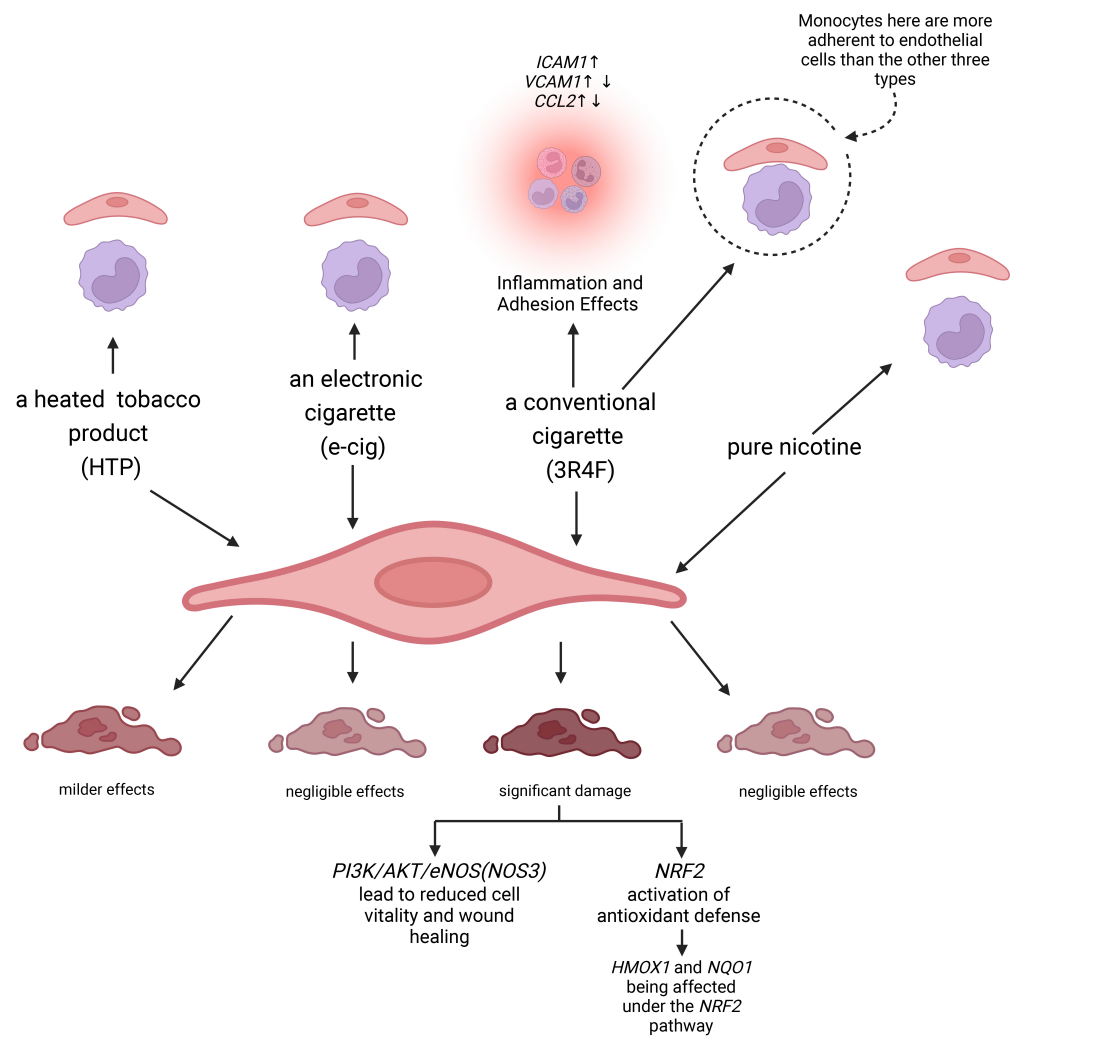
**Each tobacco product activates either an antioxidant or a pro-inflammatory response**. The responses to next-generation tobacco and nicotine products (NGPs) were generally less pronounced than in cells exposed to traditional cigarettes (3R4F). Moreover, stimulation by conventional cigarettes (3R4F) led to impaired endothelial wound healing and induced a pro-inflammatory phenotype, as opposed to the milder effects observed with NGP treatment. *PI3K*, phosphoinositide 3-kinase; *AKT*, protein kinase B; *eNOS (NOS3)*, endothelial nitric oxide synthase; *HMOX1*, heme oxygenase 1; *NQO1*, NAD(P)H quinone dehydrogenase 1; *ICAM1*, intercellular adhesion molecule 1; *VCAM1*, vascular cell adhesion molecule 1; *CCL2*, chemokine (C-C motif) ligand 2; *NRF2*, nuclear factor erythroid 2-related factor 2.

Surveys indicate that various smoking products, such as a heated tobacco product (HTP), electronic cigarettes (e-cig), traditional cigarettes (3R4F), and pure nicotine, all exert varying degrees of impact on endothelial function and dysfunction. The study found that traditional cigarettes (3R4F) significantly impair endothelial cell vitality and wound healing through the PI3K/AKT/eNOS (NOS3) (phosphoinositide 3-kinase/protein kinase B/endothelial nitric oxide synthase) pathway. Additionally, 3R4F activates the nuclear factor erythroid 2-related factor 2 (NRF2) (*NRF2*) antioxidant defense system and affects the expression of genes like heme oxygenase 1 (*HMOX1*) and NAD(P)H quinone dehydrogenase 1 (*NQO1*). While HTP demonstrated similar but milder effects, the impact of e-cig and nicotine exposure was negligible. Inflammation response analysis showed that 3R4F treatment increased intercellular adhesion molecule 1 (*ICAM1*) expression and had varying effects on vascular cell adhesion molecule 1 (*VCAM1*) and chemokine (C-C motif) ligand 2 (*CCL2*) expression. Under static conditions, all smoking products increased monocyte adhesion to endothelial cells, with 3R4F showing pronounced effects under low-flow conditions. In conclusion, while all products triggered antioxidative or pro-inflammatory responses to some extent, cells exposed to next-generation tobacco and nicotine products (NGPs) were less affected compared to those exposed to 3R4F [56] (Fig. 1).

#### 2.2.3 The Relationship between Hypertension and Cardiovascular Diseases 

Cardiovascular diseases are a leading cause of global mortality and disability, with hypertensive patients being a high-risk group for such conditions [57, 58]. Hypertension is a common disease encountered in primary care and has a causal relationship with cardiovascular diseases. With the introduction of a higher threshold for diagnosing hypertension, an increase in its reported prevalence is expected, consequently exposing more individuals to the risk of cardiovascular diseases [59]. Apart from increasing the likelihood of arterial hypertension, smoking also elevates the risk of other cardiovascular diseases in passive smokers with hypertension [60]. Numerous prospective cohort studies have indicated that hypertension is a significant risk factor for overall mortality and cardiovascular diseases [61]. Moreover, hypertension is a primary risk factor for stroke, myocardial infarction, and heart failure, collectively responsible for one-third of global deaths [62]. Hypertension is also one of the most prevalent comorbidities in heart failure and left ventricular dysfunction populations, underlining the importance of hypertension control in these groups [63]. Smoking induces inflammation, stimulates central nervous system sympathetic activity, and leads to metabolic alterations, which are critical risk factors for endothelial dysfunction and hypertension [64, 65, 66]. Hypertensive patients exposed to secondhand smoke (SHS), especially those with high body mass index, exhibit increased epicardial adipose tissue thickness, which in turn increases the risk of cardiovascular disease [67].

Heart diseases, including coronary heart disease, heart failure, atrial fibrillation, valvular disease, sudden cardiac death, sick sinus syndrome, and cardiomyopathy, are all associated with hypertension. Multivariate analysis reveals a substantial population-attributable risk of hypertension for heart failure, accounting for 39% of cases in males and 59% in females [68]. Among hypertensive individuals, myocardial infarction, diabetes, left ventricular hypertrophy, and valvular heart disease are predictors of increased risk of heart failure in both genders [69]. Prolonged hypertension ultimately leads to heart failure, and thus the majority of heart failure patients have a history of hypertension. Preventing and lowering hypertension may contribute to reducing the risk of heart disease. Research suggests that chronic pressure overload contributes to the development of left ventricular hypertrophy. Progressive hypertrophy and fibrotic changes in the heart lead to diastolic dysfunction and eventually elevated left filling pressures and diastolic heart failure [70]. Investigative results indicate that the risk of carotid atherosclerotic plaques in hypertensive patients is related to the initial intima-media thickness rather than the type of blood pressure control or antihypertensive treatment, implying challenges in achieving treatment strategies to halt vascular disease progression once vascular damage is established [71]. Individuals who smoke with hypertension or high serum cholesterol require special attention to prevent cardiovascular disease-related mortality. Hypertensive smokers often experience poorer outcomes, such as coronary artery and cerebral infarction mortality [72]. A meta-analysis of individual patient data underscores the inseparable relationship between hypertension and heart disease, suggesting similar relative protection across all baseline cardiovascular risk levels through blood pressure reduction, which lowers the risk of cardiovascular diseases [73].

### 2.3 Arteriosclerosis and Coronary Heart Disease

#### 2.3.1 The Impact of Smoking on Arteriosclerosis

CVD arising from atherosclerosis of arterial vessel walls and thrombus formation are the leading cause of premature death and disability-adjusted life years (DALYs) in Europe and are increasingly prevalent in developing countries [4]. Smoking alters the coronary artery vascular tone, affects platelet activation and endothelial integrity, and promotes the occurrence and progression of atherosclerosis. Globally, approximately 1 billion men and 250 million women currently use tobacco, with smoking rates and tobacco consumption increasing yearly [74]. Numerous studies indicate that smoking can induce endothelial dysfunction related to atherosclerosis, cerebrovascular diseases, coronary artery diseases, and hypertension [25]. Atherosclerosis and associated cardiovascular diseases are among the most common causes of death and morbidity in Western societies, with over 10% of cardiovascular disease deaths attributed to smoking [33]. Research demonstrates that active smoking and exposure to secondhand smoke are associated with the progression of the atherosclerosis index. The impact of smoking on the speed of atherosclerosis progression is cumulative and irreversible. Smoking-induced microcirculatory pathological changes often involve moderate narrowing of the vessel lumen and thickening of the vessel wall. Over time, these microvascular pathologies cause significant damage primarily affecting the heart, brain, and kidneys [43]. Tobacco smoke contributes to thrombus formation and accelerates atherosclerosis, increasing the risk of acute myocardial infarction, sudden cardiac death, stroke, aortic aneurysm, and peripheral vascular disease. Even extremely low levels of exposure can increase the risk of acute myocardial infarction. Moreover, nicotine in tobacco products can accelerate the intimal thickening of the femoral artery following balloon catheter injury and potentially promote atherosclerosis [16].

The state of atherosclerosis induced by smoking and the development and progression of vascular injury are inextricably linked [75]. Various studies have demonstrated that smoking induces oxidative stress, vascular inflammation, platelet coagulation, vascular dysfunction, and impairs blood lipid levels in current and long-term smokers as well as in active and passive smokers, resulting in adverse effects on the cardiovascular system [10]. A recent study has shown that the smoke from tobacco during smoking accelerates calcium deposition in arteries, increasing the rate of atherosclerosis by 10% to 20%, thus raising the risk of heart attacks and strokes [76]. Female smokers have a 25% higher risk of ischemic heart disease than male smokers, possibly due to the influence of smoking on menopause and its anti-estrogenic effects, with long-term smoking having a greater impact on atherosclerosis in women than in men [77]. According to multiple cohort studies, case-control studies, and meta-analyses, secondhand smoke increases the risk of coronary heart disease by 25–30% [78]. Physiological and basic science research indicates that the mechanisms through which secondhand smoke affects the cardiovascular system are diverse, including increased thrombosis and low-density lipoprotein oxidation, reduced exercise tolerance, impaired flow-mediated dilation, and activation of inflammatory pathways associated with oxidative damage and compromised vascular repair. Therefore, chronic exposure to secondhand smoke promotes the development of atherosclerosis and cardiovascular disease, increasing the risk of acute coronary syndromes [78]. Multiple research reports suggest that exposure to secondhand smoke is associated with increased intima-media thickness of the carotid artery and coronary artery calcification, leading to numerous irreversible harms to our bodies [52, 79, 80]. Hence, smoking cessation and the implementation of smoking bans are crucial for reducing the risk of atherosclerosis, protecting cardiovascular health, enhancing immune responses, and prolonging human life.

#### 2.3.2 The Connection between Active/Passive Smoking and Heart Failure and Atrial Fibrillation

Heart rate variability (HRV) is a direct and cost-effective technique for predicting health issues related to cardiovascular properties and can foresee the impact of smoking on health. The majority of published studies indicate that both acute and chronic active and passive smoking significantly disrupt the normal functioning of the autonomic nervous system. This disruption is characterized by increased sympathetic nerve drive, reduced HRV, and diminished parasympathetic regulation [81]. A few studies suggest that exposure to SHS correlates with poorer quality of life and higher all-cause mortality among heart failure (HF) patients who are non-active smokers. Recent research shows that in non-active smokers, a moderate increase in urinary cotinine levels (greater than 7.07 ng/mL) is associated with a 40–50% increased risk of any heart failure events [82]. Both active and passive smoking are potential contributors to the onset of heart failure [83].

Prolonged atrial fibrillation can lead to heart failure, and recurrent heart failure can cause atrial enlargement and further atrial fibrillation. Research indicates an association between smoking and an increased risk of atrial fibrillation, which is dose dependent. In never-smokers, higher exposure to SHS, as objectively measured by urinary cotinine levels, may be associated with a 1.6-fold increased risk of new-onset atrial fibrillation [84]. In summary, both active and passive smoking have the potential to cause heart failure and atrial fibrillation.

#### 2.3.3 Association between Smoking and Coronary Heart Disease

Coronary artery atherosclerotic heart disease is a condition in which atherosclerotic lesions occur in the coronary arteries, leading to the narrowing or blockage of the vessel lumen, resulting in myocardial ischemia, hypoxia, or necrosis, and thus causing heart disease, commonly referred to as “coronary heart disease”. Experimental data indicate that 50% of long-term smokers die from tobacco-related diseases, with heart disease being a primary cause of mortality among smokers [6]. Studies suggest that light smokers have a 65% higher likelihood of developing coronary heart disease compared to individuals with no smoking history, and the risk of coronary heart disease is even greater for long-term smokers [85].

Active and passive smoking remain global epidemics and critical risk factors for the development of cardiovascular diseases. The cardiac damage induced by active and passive smoking involves two main and interchangeable mechanisms: one is the direct adverse response on the myocardium leading to smoking-related cardiomyopathy, and the other is the indirect impact on the myocardium through the promotion of complications such as atherosclerosis and hypertension, ultimately causing damage and remodeling of the heart [86]. Among patients with acute coronary syndrome, smokers have a higher rate of thrombus formation within stents compared to nonsmokers, and more than two-thirds of smokers die from sudden cardiac death caused by acute atherosclerotic thrombosis [33, 87]. Smoking not only affects blood coagulability and hinders blood flow but is also closely associated with carotid intima-media thickness, atherosclerotic diseases, and the progression of carotid intima-media thickness [88]. Moreover, even without active smoking habits, exposure to secondhand smoke is a contributing factor to increased global morbidity and mortality rates. Secondhand smoke is an established cardiovascular disease risk factor, with one-third of nonsmoking adults worldwide being exposed to it [89, 90]. Previous research has demonstrated that secondhand smoke exposure may increase the risk of cardiovascular diseases by influencing inflammatory and atherosclerotic pathways [91, 92]. Meta-analyses estimate that secondhand smoke exposure is associated with a 31% increased risk of coronary heart disease and a 20–30% increased risk of stroke [2, 3, 92].

**Fig. 2.**
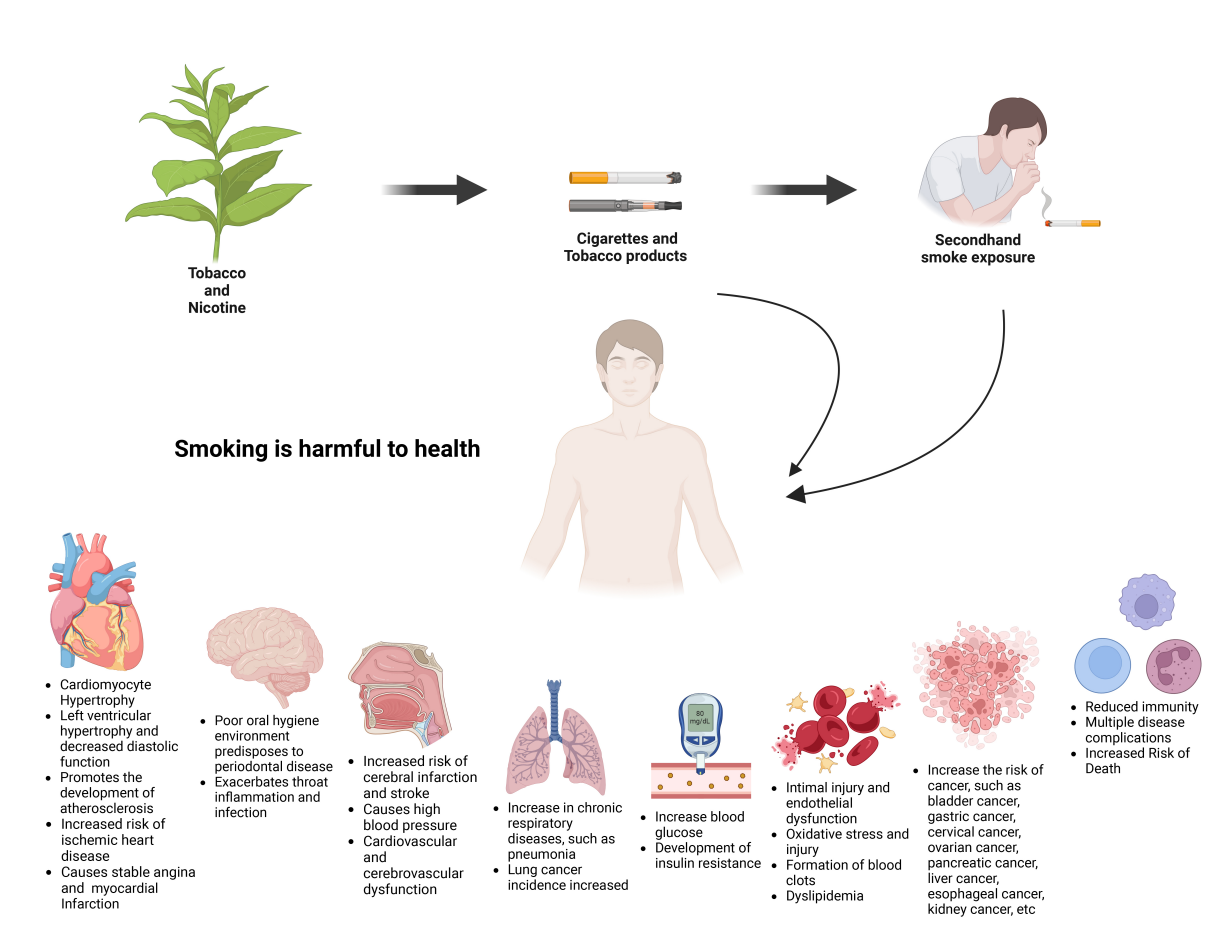
**Smoking is harmful to health**. Long-term smoking or exposure to secondhand smoke can easily cause diseases of the cardiovascular system, nervous system, respiratory system and digestive system.

Tobacco use is a well-recognized risk factor for the incidence and mortality of cardiovascular diseases, increasing the risk of death from all vascular diseases by 2 to 3 times [6, 93, 94]. Globally, 10–30% of cardiovascular disease deaths can be attributed to smoking. However, among males aged 30 to 44 years, 48% of cardiovascular deaths are attributable to smoking. The risk of cardiovascular diseases among smokers increases with the number of cigarettes smoked per day [94]. Among the various diseases caused by smoking, similar to the established impact of smoking on subclinical atherosclerosis, coronary artery calcification is more prevalent in individuals who have never smoked but are exposed to secondhand smoke compared to those who are unexposed to secondhand smoke and have never smoked [51]. The effects of smoking on the cardiovascular system and coronary risk factors are pervasive. Detrimental effects include acute increases in blood pressure and coronary vascular resistance, reduced oxygen delivery, enhanced platelet aggregation, elevated fibrinogen levels, and decreased high-density lipoprotein cholesterol. Secondhand smoke exposure can lead to dose-dependent increases in mortality among heart failure patients, elevated incidence of coronary heart disease among non-smokers exposed to secondhand smoke during childhood, increased risk of atrial fibrillation in adulthood, and more [95, 96, 97]. Tobacco is often the forgotten risk factor for heart disease. It is crucial to recognize that smoking shares characteristics of chronic diseases and should be treated as such (Fig. 2).

## 3. Smoking Cessation and Heart Health

### 3.1 Benefits of Quitting Smoking. 

#### 3.1.1 Recommendations for Heart Health

The Heart is a Tirelessly Functioning Organ, Working Ceaselessly from the Embryonic Stage of Human Life. Regular check-ups are one of the simplest means to detect and prevent heart disease, as they help in screening for risk factors like hypertension, diabetes, and hyperlipidemia. Additionally, quitting smoking, maintaining a healthy and balanced diet, fostering a positive and optimistic outlook on life, routinely monitoring blood pressure, managing cholesterol and blood glucose levels, and maintaining a healthy weight all contribute to safeguarding heart health. Sufficient sleep plays a pivotal role in reducing stress, enhancing emotional well-being, sharpening alertness, replenishing energy, and improving heart health. Ensuring high-quality sleep can help mitigate the risk of heart attacks. Regular physical exercise positively affects the cardiovascular system, improving cardiopulmonary health and offering protective effects for the heart [98].

#### 3.1.2 Potential Benefits of Reducing Heart Disease Risk

The “State of Tobacco Control 2024” report by the American Lung Association underscores the detrimental impact of tobacco use, including menthol cigarettes, across the United States. It calls for the final formulation of regulations to cease the sale of menthol cigarettes and flavored cigars, a critical step in saving lives.

**Fig. 3.**
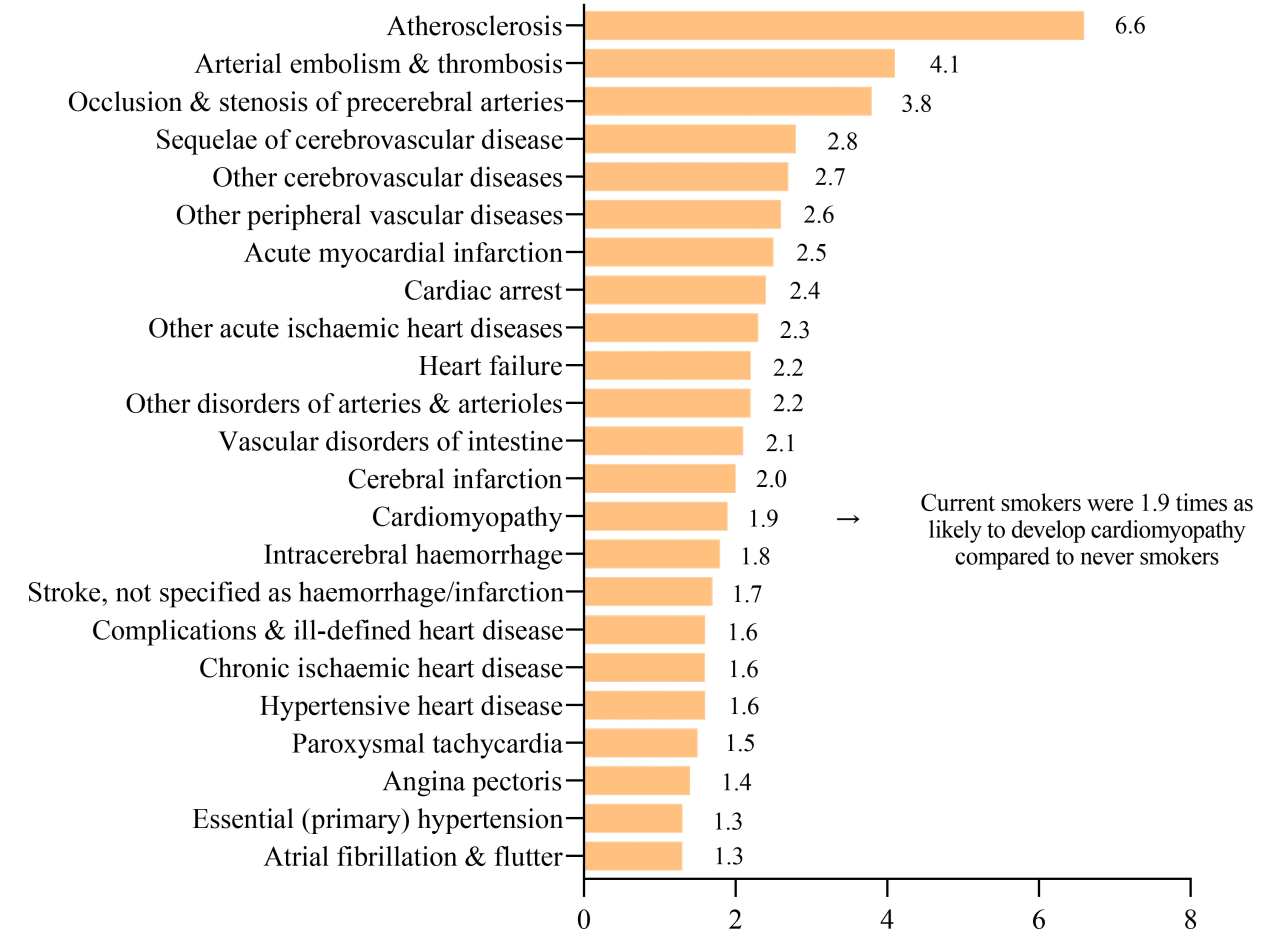
**illustrates the relative risks for specific CVD subtypes, indicating how many times more likely current smokers are to develop these CVD subtypes compared to non-smokers, with the increased risk in current smokers being statistically significant**. CVD, cardiovascular diseases.

There is a marked disparity in the mortality rates from smoking-related CVD between different countries, influenced primarily by smoking rates, public health policies, and healthcare systems. In high-income countries like the United States and Western Europe, including Germany, Greece, Hungary, Poland, Romania, and Spain, smoking rates have declined over the past decade due to stringent anti-smoking laws, effective public health campaigns, and increased awareness [99]. This decline is paralleled by a gradual decrease in smoking-related CVD mortality rates. However, smoking remains a significant cause of CVD, particularly in lower socio-economic groups and more so in men than in women. In contrast, Asian and Eastern European countries report high smoking-related death rates, while low-income countries with lower smoking prevalence report much lower rates [100]. In China and Russia, higher CVD mortality rates are linked to smoking. Public health interventions and smoking bans are necessary, as demonstrated by the decline in ischemic heart disease (IHD), acute myocardial infarction (AMI), and CVD deaths across all age groups and those over 65 in Hong Kong following the implementation of smoke-free policies [101]. A significant study from Australia indicates that smokers face an elevated risk for most types of CVDs [102]. The study found that CVD mortality rates were nearly threefold higher in current smokers compared to individuals who have never smoked. Smoking accounts for 15% of all cardiovascular deaths across various age groups in Australia, with a notably higher proportion in middle-aged individuals. Specifically, for those aged 45 to 54, cardiovascular mortality rates stood at 38.2% for men and 33.7% for women. Smoking emerges as a strong, independent risk factor for increased cardiovascular disease and mortality, a trend persisting even into older age groups. Notably, smokers older than 60 years exhibit a cardiovascular mortality increase exceeding five years. Furthermore, heavy smoking, defined as 25 or more cigarettes per day, escalates the risk of CVD-related death nearly fivefold. Even light smoking, categorized as 4–6 cigarettes per day, almost doubles the risk of CVD mortality [103]. A major Australian study conducted in 2019 concluded that current smoking significantly increases the risk for nearly all subtypes of CVD, with many subtypes seeing at least a doubling of risk [102] (Fig. 3).

Concerningly, older populations typically exhibit higher smoking-related CVD mortality rates due to the cumulative effects of smoking, while increasing smoking prevalence among adolescents and young adults poses long-term public health challenges. Exposure to secondhand smoke in childhood has been shown to adversely affect cardiac arterial function and structure, leading to a higher risk of atherosclerosis and cardiovascular diseases [104]. This exposure is also associated with impaired cardiac autonomic function and changes in heart rate variability. Notably, gender differences are evident, with higher smoking rates and consequent CVD mortality in men. However, rising smoking rates among women in many regions may lead to an increase in female CVD mortality rates in the coming years.

Smoking cessation offers the potential to decrease the risk of lung cancer, chronic obstructive pulmonary disease, heart disease, and numerous other chronic ailments [105]. Smoking remains a leading preventable cause of morbidity and mortality. Among smokers, complete smoking cessation is the most effective approach to preventing cancer and cardiovascular diseases. Prolonged smoking leads to various irreversible cardiovascular pathologies, adversely affecting cardiovascular health [106, 107, 108]. Research shows that quitting smoking can improve high-density lipoprotein cholesterol, overall high-density lipoprotein levels, and large high-density lipoprotein particles, especially in females [109]. Smoking cessation helps reduce the incidence of heart disease and cardiac events, while both abstaining from smoking, and avoiding secondhand smoke exposure, contribute to mitigating cardiovascular-related health complications, rectifying smoking-associated physiological abnormalities [110, 111]. Moreover, smoking cessation can improve existing vascular inflammation and damage. Premature atherosclerosis has been noted in young smokers, to be substantially improved upon quitting. Therefore, prompt smoking cessation is crucial, as reversing vascular damage may become infeasible over time [112].

### 3.2 Smoking Cessation Methods and Resources

#### 3.2.1 Medical Assisted Smoking Cessation Therapy

Smoking is a chronic condition driven by both physical nicotine dependence and learned behavior. Due to nicotine’s highly addictive nature, unassisted smoking cessation rates remain remarkably low. Approximately 70% of smokers wish to quit, and those attempting to quit often need about 6 quit attempts before achieving long-term abstinence [113]. Currently, the most effective smoking cessation treatments involve a combination of behavioral counseling and pharmacotherapy, which yield significant success rates. Recommended behavioral interventions include advice from physicians, nurses, or smoking cessation specialists, as well as counseling via telephone or mobile-based applications. The U.S. Food and Drug Administration (FDA) has approved three medications for nicotine dependence treatment: Varenicline (oral); Nicotine Replacement Therapy (transdermal patches, gum, lozenges, inhalers, or nasal sprays); Bupropion Hydrochloride Sustained-Release (oral). The standard treatment duration for smoking cessation medications is typically 6 to 12 weeks, with the option to extend beyond 12 weeks to enhance cessation rates [105].

#### 3.2.2 The Importance of Psychological and Behavioral Support

The urgency of implementing strong tobacco control policies is underscored by the substantial health and economic burdens of tobacco use globally. According to the World Health Organization (WHO), effective tobacco control policies, including increased tobacco taxes and comprehensive smoke-free policies, can generate significant government revenues and reduce tobacco use, ultimately protecting public health from diseases like cancer and heart disease. For instance, a 20% price increase on tobacco products could lead to substantial healthcare cost savings and reduced productivity losses. Comprehensive smoke-free policies are shown to improve indoor air quality, reduce exposure to secondhand smoke, and lower the prevalence of tobacco smoking, in addition to reducing deaths and hospitalizations from cardiovascular and respiratory diseases.

The WHO’s 2021 report on the global tobacco epidemic presented new data on electronic nicotine delivery systems (e.g., e-cigarettes), which are often marketed to children and adolescents using appealing flavors and misleading claims. The report recommends that governments implement regulations to stop non-smokers from starting to use these products, to prevent the renormalization of smoking in the community, and to protect future generations. Currently, 32 countries have banned the sale of electronic nicotine delivery systems, and a further 79 have adopted at least one partial measure to regulate these products.

Given the high global and regional variability in tobacco use and the corresponding health effects, with approximately 80% of the world’s smokers living in low- and middle-income countries, these measures are especially critical. The WHO has emphasized the need for concerted efforts to ensure that progress in tobacco control is maintained or accelerated.

Implementing strong tobacco control measures, such as raising tobacco taxes and enforcing comprehensive smoke-free policies, is thus a critical step in reducing the global burden of tobacco-related diseases and deaths. These strategies not only improve public health but are also cost-effective and do not harm economies.

**Fig. 4.**
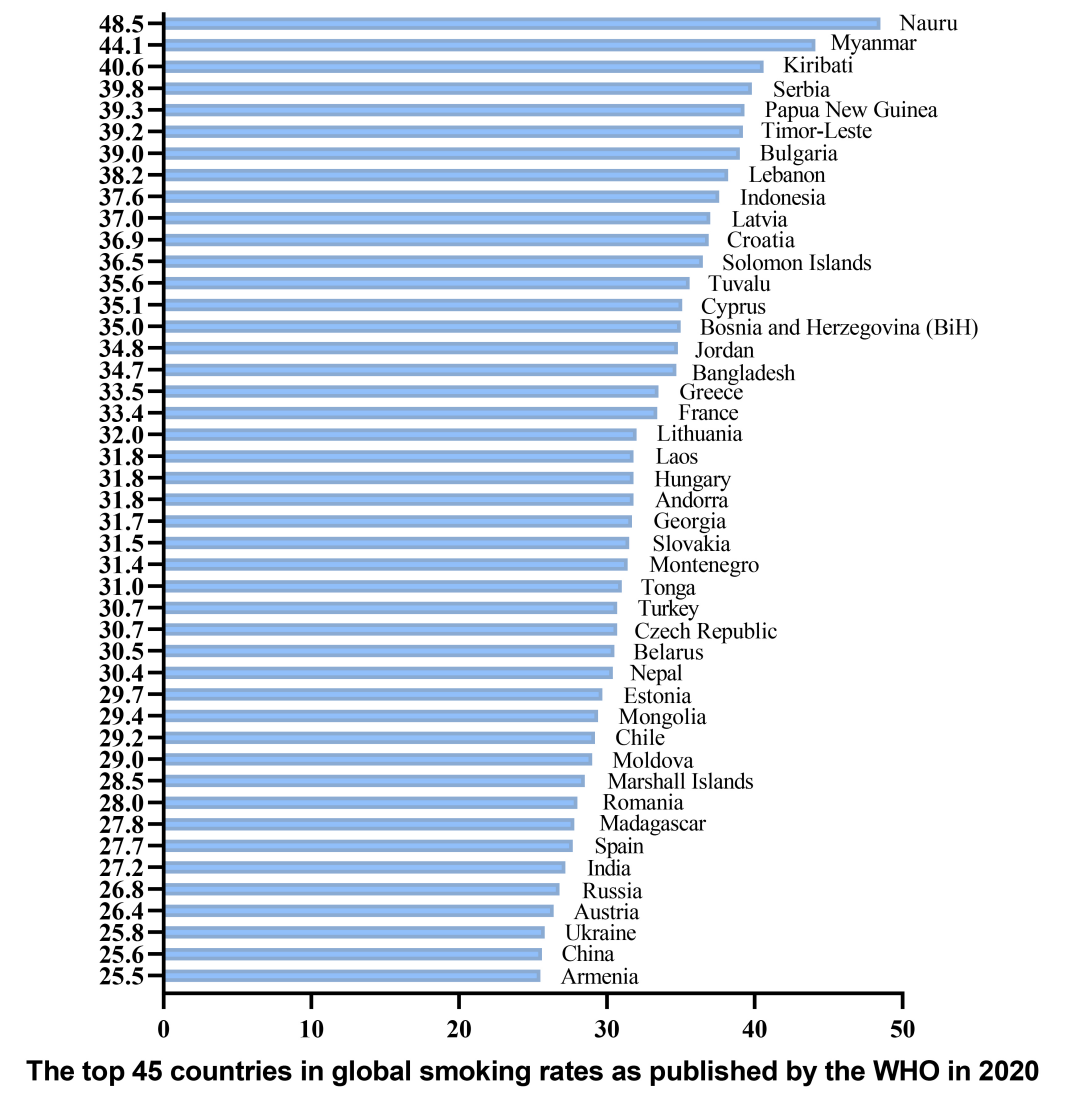
**The top 45 countries in global smoking rates as published by the WHO in 2020**. WHO, the World Health Organization.

Governments should enhance public awareness of cardiovascular diseases induced by cigarette smoke and encourage individuals to reduce their exposure to cigarette smoke, thereby mitigating the adverse consequences related to atherothrombotic diseases [33]. In terms of smoking cessation, psychological and behavioral support plays a pivotal role. On one hand, based on psychological and sociological models, an emphasis is placed on reinforcing self-control and utilizing behavior change theories are emphasized. On the other hand, medical models focus on managing withdrawal symptoms to promote successful cessation, primarily employing nicotine replacement therapy. However, it is evident that products like electronic cigarettes still pose health risks, so quitting and completely abstaining from smoking is preferable. The health benefits of quitting smoking are more pronounced for those who quit earlier, but indeed, quitting smoking at any age is advantageous for overall health. In many countries, the prevalence of smoking among adolescents is high, yet the majority of these smoking students express a desire to quit. Among adults, there is a significant smoking population, but the proportion actively willing to quit actively is lower, primarily due to limited awareness of smoking hazards and inadequate knowledge about cessation methods. Consequently, the utilization of medication-based smoking cessation methods is low among those opting for cessation methods. Research indicates that the most effective measure in protecting individuals from the harm of tobacco smoke involve government-led behavioral interventions. Smoke-free policies are associated with reduced smoking behavior, secondhand smoke exposure, and numerous adverse health outcomes [114]. Additionally, increasing taxes on tobacco products also contributes to reducing smoking behavior. Simultaneously, governments can encourage smoking cessation by implementing pictorial cigarette warning labels [115]. This article presents a chart depicting the top 45 countries in global smoking rates as published by the WHO in 2020 (Fig. 4).

## 4. Conclusions

The impact of smoking on CVD remains a critical area of medical research. It is well-established that smoking is one of the leading preventable causes of CVD, which includes coronary heart disease, peripheral arterial disease, stroke, and heart failure. The harmful substances in tobacco smoke, particularly nicotine, carbon monoxide, and various oxidants, play a significant role in damaging the cardiovascular system. These substances contribute to the development of atherosclerosis, characterized by the buildup of fatty substances in the arteries, leading to reduced blood flow and increased risk of clot formation.

Nicotine, a key active component in tobacco, has been shown to increase heart rate and blood pressure, contributing to a heightened workload on the heart and promoting arterial stiffening and narrowing. Furthermore, smoking has been linked to the disruption of normal endothelial function, a condition vital for maintaining vascular health. The endothelial dysfunction caused by smoking contributes to inflammation and plaque formation within arteries, significantly elevating the risk of acute cardiovascular events, such as myocardial infarction.

Additionally, research has underscored the importance of quitting smoking for heart health. Smoking cessation has immediate and long-term cardiovascular benefits, reducing the risk of CVD significantly over time. Within a year of quitting, the risk of coronary heart disease drops substantially and continues to decline thereafter. The beneficial effects of smoking cessation have been observed even among individuals with existing CVD. Notably, quitting smoking can reverse some of the endothelial damage and improve vascular function, highlighting the importance of smoking cessation interventions in public health and clinical settings.

This review provides a brief overview of recent insights into the adverse effects of smoking on cardiovascular health and the substantial benefits of smoking cessation. Smoking cessation should be an integral part of our preventive and public health strategies in cardiology.
